# SARS-CoV-2 vaccination in children and adolescents with and without type 1 diabetes mellitus

**DOI:** 10.1007/s12020-023-03471-y

**Published:** 2023-08-16

**Authors:** Kyriaki Karavanaki, Spyridon Karanasios, Alexandra Soldatou, Maria Tsolia

**Affiliations:** Diabetes and Metabolism Clinic, 2nd Department of Pediatrics, National and Kapodistrian University of Athens, “P&A Kyriakou” Children’s Hospital, Athens, Greece

**Keywords:** COVID-19, Pediatric, Immunization

## Abstract

Adults with Diabetes Mellitus (DM) have increased risk of severe clinical presentation during COVID-19 infection, while children and adolescents with type 1 diabetes (T1D) have the same mild clinical course as their healthy peers, especially those with optimal glycemic control. The present review focuses on the necessity of COVID-19 vaccination among children and adolescents with T1D, and also in their non-diabetic peers. The efficacy and safety of COVID-19 vaccines are also discussed, as well as their various side-effects, ranging from common mild to very rare and serious ones. Furthermore, the results of COVID-19 vaccination of adolescents with and without T1D are reported, as well as the efficacy and concerns about childhood vaccination. It is concluded that patients with DM of all age groups should maintain optimal diabetic control in order to avoid glycemic deterioration during COVID-19 infection. Furthermore, despite the very rare and serious complications of COVID-19 vaccines, vaccination against COVID-19 is recommended for children and adolescents with T1D to prevent glycemic deterioration and rare but serious complications of COVID-19 infection.

## Introduction

Coronavirus disease 19 (COVID-19) first presented in Wuhan, China, and was attributed to severe acute respiratory syndrome Coronavirus 2 (SARS-COV2). In March 2020, WHO categorized COVID-19 as a pandemic, necessitating the introduction of a variety of restrictive measures to prevent contamination. Countermeasures included sanitary precautions (e.g., surgical masks, social distancing, regular hand-washing and antiseptic use), viral detection tests (e.g., antigen tests, PCR), self-isolation, lockdowns, and immunization programs [1]. As a result, a significant decline in cases of COVID-19 was observed. However, severe health, psychosocial and economic repercussions of these measures were reported, while new variants have emerged propagating subsequent waves of the pandemic. As of September 2021, new vaccines against COVID-19 have been introduced and the global immunization program is considered one of the most effective measures to mitigate the pandemic.

### Measures for severe infection prevention in patients with diabetes mellitus

Patients with diabetes mellitus (DM) have been categorized among those with other chronic conditions, as a high-risk group for severe COVID-19 infection. Vaccination against SARS-CoV-2 to prevent COVID-19 infection is recommended, as well as for the H1N1 influenza virus. Moreover, optimal glycemic control, use of continuous glucose monitoring (CGM) devices for time in range (TIR) estimation and telemedicine for maintaining regular follow-up by the Diabetes team during lockdown periods, were implemented [2].

### Types of COVID-19 vaccines

To date, there are seven different types of vaccines against COVID-19 approved by the Food and Drug Administration (FDA), based on four different technologies, described in Table 1 [2]. Among them, mRNA vaccines have the highest success rates (94.1–95%), followed by the adenovirus-vector vaccines (70.4–91.6%) and vaccines with protein subunit of nanoparticles (Novavax) (89.3%). The inactivated vaccine CoronaVac from China has a variable success rate, ranging between 50.6–91.2% [2].

**Table 1:** Types of COVID-19 vaccines [2]

Vaccine Name	Type	Success Rate	Complications
ΒΝΤ16b2 (Pfizer-BioNTech)	mRNA vaccine → codes for a protein spike	95%	Localized pain, fatigue, headache Rare: myocarditis, pericarditis, anaphylaxis
mRNA-1273 (Moderna)	mRNA vaccine → codes for a protein spike	94.1%	Temporary localized and systematic symptoms Rare: myocarditis, pericarditis, anaphylaxis
AZD1222 (Oxford- Astra Zeneca)	Recombinant, chimpanzee adenovirus-vector vaccine, with a protein spike antigen	70.4%	Severe complications in very few cases
Sputnik V vaccine (Gamaleya Research Institute, Russia)	Recombinant, adenovirus-vector vaccine rAd, with a protein spike antigen	91.6%	Flu symptoms, localized reactions, fatigue, headache, no severe complications
JNJ-78436735 (Johnson & Johnson, Janssen Biotech Inc)	Recombinant, adenovirus-vector Ad26 vaccine, encoding spike protein	66%	Fever (9%) with no severe complications Rare: Severe allergic reactions, anaphylaxis
Novavax, Inc USA	Recombinant, with a protein subunit of nanoparticles, wild type of protein spike	89.3%	Severe complications in very few cases Rare: myocarditis, pericarditis, anaphylaxis
CoronaVac (Sinovac Biotech China)	Inactivated vaccine	50.65–91.25%	NA

## The necessity of covid-19 vaccination for patients with diabetes mellitus

It has been reported that patients with DM are more vulnerable to COVID-19 infection [3]. Indeed, in patients with DM, neutrophil and natural immunity functions are compromised. Specifically, patients suffering from type 1 diabetes (T1D), have a higher chance of being infected by SARS-CoV2, than those with type 2 diabetes (T2D). Additionally, poor glycemic control, inevitably leads to the glycation of the angiotensin-converting enzyme 2 (ACE2) receptors, where SARS-CoV-2 binds to the cell membrane, making poorly controlled DM patients more prone to COVID-19 infection [4].

On the other hand, COVID-19 infection leads to glycemic derangement with severe hyperglycemia and metabolic deterioration as a result of increased insulin resistance associated with increased levels of pre-inflammatory cytokines, such as TNF-a, IL-1b and IL-6b [5]. Moreover, during COVID-19 infection a decrease in insulin secretion is also observed, due to the secretion of counteracting hormones and the activation of the Renin-Angiotensin-Aldosterone (RAAS) system, effectively destroying pancreatic β-cells, which leads to newly diagnosed diabetes. Lastly, insulin secretion may be inhibited due to the downregulation of ACE2 receptors, caused by SARS-CoV-2 virus [6].

In fact, Boddu SK et al. [3] have reported a bidirectional association between COVID-19 and diabetes pandemics. Thus patients with DM with poor glycemic control are more vulnerable to COVID-19 infection. This leads to further metabolic derangement, several complications and increased mortality. On the other hand, due to the direct effect of SARS-COV-2 on pancreatic β-cells, patients with COVID-19 may develop hyperglycemia, acute destruction of pancreatic β-cells, resulting to newly diagnosed diabetes with increased mortality [3, 7].

### Severity of COVID-19 infection in children

It has been substantially proven that COVID-19 infection in adult patients suffering from diabetes incurs increased morbidity and mortality rates. Moreover, DM patients suffered from an overall more severe clinical course of the disease, even presenting with multi-organ failure [8]. Children have the same chances to be infected by the SARS-CoV-2 virus, as adults. However, children, adolescents, and young adults (under 25 years of age) with T1D, usually have a milder COVID-19 infection course than adults with DM and are not at increased risk for hospitalization [9, 10], have a shorter duration of disease and a highly favorable prognosis with rare deaths [11, 12].

On the other hand, infants and children with underlying conditions are at a higher risk of presenting with severe COVID-19 infection [13]. Serious complications among children and adolescents include multisystem hyperinflammatory syndrome (MIS-C), myo-/pericarditis, long COVID-19 syndrome, and very rarely Guillain Barre syndrome [14]. A study from Denmark reported that 0.49% of children with positive RT-PCR required hospital admission, 0.01% ICU admission and 0.05% developed MIS-C within 2 months of COVID-19 infection. There were only 5 cases with Guillain Barre syndrome and none with myocarditis or encephalitis [15].

### Risk factors for severe pediatric COVID-19 infection

As mentioned above, age plays a critical role in the severity of infection. Specifically, infants have an increased risk of developing serious respiratory involvement (10.6%), due to the smaller diameter of the airways and the immaturity of their immune system [13].

Concerning the ethnic background, Africans and Hispanics have been reported to have a higher chance of developing ARDS. It has also been noted that male gender, pregnancy and active or passive smoking, carry an increased risk. Underlying pediatric conditions associated with severe infection [13] are shown in Table 2.

**Table 2:** Underlying pediatric conditions associated with severe COVID-19 infection

• Diabetes Mellitus (both types)
• Obesity
• Hypertension
• Immunosuppression
• Malignancies
• Neurological, genetic, and metabolic conditions
• Cardiovascular, kidney and chronic liver conditions
• Chronic respiratory problems (cystic fibrosis, asthma)
• Chronic hematological conditions (β-thalassemia, sickle cell disease).

### Frequency of COVID-19 infection in children with T1D

Studies from countries with increased prevalence of COVID-19, such as Italy, report a lower incidence of the disease among children with newly diagnosed T1D [16]. There are many explanations for this phenomenon, including the patients’ younger age, as well as concurrent protective measures (lockdown). Moreover, the decreased overall prevalence of patients with T1D and an increased expression of CD8+ lymphocytes in patients with T1D, may also explain this observation [17]. However, children with T1D, especially those with poor diabetic control, sometimes require hospital admission when they present with COVID-19 infection [18].

### Increased risk of Diabetic Ketoacidosis (DKA) due to COVID-19 in children and adolescents with T1D

In a study of 266 patients under the age of 19 years with known T1D and COVID-19 infection, 61 (22.9%) required hospitalization [19]. Of those requiring hospitalization, in 44 (72%) it was due to DKA, in 3 (4.9%) due to severe hypoglycemia, in 3 (4.9%) due to severe respiratory distress and in 1 (1.6%) due to MIS-C. Comparison of two T1D patient groups—with and without hospitalization – showed the following risk factors for hospitalization: foreign nationality and/or ethnic minority (67 vs 39%, *p* = 0.001), high HbA1c (11 vs 8.2%, *p* = 0.001) and a reduced frequency of diabetes technology use, i.e. insulin pumps/continuous glucose monitors (26 vs 54%, *p* = 0.001, 39 vs 75%, *p* = 0.001) [19]. Many previous studies report the association of COVID-19 infection with the development of DKA in patients with known as well as newly diagnosed T1D [20–23]. Glycemic deterioration during COVID-19 infection has been associated with increased insulin resistance due to COVID-19 infection [5], as well as acute destruction of pancreatic β cells due to the direct action of the virus itself, precipitating T1D development in patients with pancreatic autoimmunity or acute non-autoimmune T1D [16, 24, 25].

Conclusively, poor glycemic control, reduced use of diabetes technology and poor hospital access were shown to play an important role in severe clinical presentation and hospitalization among children with known T1D and COVID-19 infection.

### COVID-19 infection and COVID-19 vaccination glycemic effects in patients with and without diabetes

COVID-19 infection seems to cause glycemic abnormalities in patients with T1D [18, 19], but also in those without diabetes. Thus, Montefusco et al. [26] reported in 551 previously healthy subjects with COVID-19 infection the development of hyperglycemia associated with the infection. Among them, 46% presented hyperglycemia with insulin resistance and altered cytokine profile, while 27% remained normoglycemic. In the hyperglycemic group, hyperglycemia persisted for 6 months in 35% of cases, while only 2% developed overt diabetes, and in the remaining 63% hyperglycemia subsided. They also found that hyperglycemic patients in the acute phase presented elevated proinflammatory cytokine levels and especially IL-6, which correlated with fasting glucose levels. Thus, the authors concluded that COVID-19 infection was associated with various degrees of glycemic impairment, insulin resistance and b-cell dysfunction in previously healthy individuals, which may last from 2 to 6 months after recovery from COVID-19 infection and may be attributed to the proinflammatory milieu caused by the cytokine storm [26].

On the other hand, D’Addio et al. [27] reported that COVID-19 vaccination did not alter glycemic control in patients with T1D and following multivariate analysis no association between HbA1c levels and cytotoxic response to vaccination was found after adjustment for age, sex, and concomitant therapies. Therefore, it is concluded that glycemic abnormalities are linked to COVID-19 infection per se rather than vaccination.

### Conclusions on the impact of COVID-19 in patients with DM

Adults with T1D suffer from an increased risk of contracting COVID-19 infection, as well as being hospitalized in a serious clinical condition. Predictably, COVID-19 infection deteriorates glycemic control, both in adults and in children with diabetes, but has also been associated with the development of hyperglycemia in previously healthy subjects. Moreover T2D, obesity, poor metabolic control, and diabetes complications result in a more severe clinical presentation of COVID-19 infection. As previously discussed, children suffer from milder clinical course of the disease, except for those belonging to high-risk groups. However, all children and adolescents with COVID-19 may present with several severe clinical conditions, including MIS-C, myocarditis and Long COVID-19 syndrome. Although young patients with T1D present with a milder clinical course of COVID-19, they have an increased risk of developing DKA, especially those with pre-existing poor diabetic control. In order to prevent such an outcome, excellent diabetic control has to be maintained.

## The effectiveness of different Covid-19 vaccines

The effectiveness of the 4 broadly available COVID-19 vaccines ranges from 73.1–94.3% (Table 3a). The highest effectiveness has been shown for mRNA and protein subunit vaccines, and the lowest for inactivated vaccines. Among the factors associated with higher vaccination effectiveness are male gender, age younger than 55 years and African/Black race (Table 3b) [2].

**Table 3:** Vaccination against COVID-19 in patients with T1D

a. Vaccination effectiveness against COVID-19 in patients with T1D:	
Vaccine Type	Effectiveness (vs placebo)
mRNA (*n* = 48.500)	94.3%
Protein Subunit (*n* = 7.500)	89.3%
Adenovirus Vector (*n* = 40.250)	79.5%
Inactivated (*n* = 5.700)	73.1%

b. Factors associated with vaccination effectiveness in patients with T1D		
	Vaccination Group	Effectiveness
Gender	Males vs Controls	92.7%
	Females vs Controls	87.8%
Age	16–55 years	88.9%
	>55 years	87.6%
Ethnic background	African/African Americans	93.4%
	Caucasian	89.8%

The Pfizer-BioNTech vaccine has been recommended by the FDA for use in adolescents aged > /12 years, administered as two doses of 30 μg, 0.3 ml each [28]. In November 2021, the FDA also issued a recommendation to use a new formulation of the Pfizer-BioNTech vaccine in children aged 5–11 years, to be administered as two doses of 20 μg, 0.2 ml each [28]. Among healthy adolescents who received the mRNA vaccine, the reported vaccine effectiveness after the first dose was 62% and after the 2^nd^ dose 93% [15]. Vaccinations against SARS-CoV2 achieve the same results for children and adults with T1D and their healthy counterparts; better protection against the virus and, if infected, a smoother clinical course [29]*.*

### Effectiveness of COVID vaccination in patients with T1D

Patients with T1D have been shown to have impaired cellular immune response following vaccination against influenza, rotavirus and Haemophilus influenza type B [30, 31]. Similarly, D’Addio et al. [27] reported that T1D patients showed in their majority, impaired cytotoxic immune response against SARS-CoV-2 after 2 doses of mRNA vaccines, while their humoral response was unaffected. The altered immune cellular response was confirmed by reduced levels of interleukin-2 (IL-2) and tumor necrosis factor-α (TNF-α) in patients with T1D after vaccination, which was associated with a low IFN-γ response, regardless of the type of mRNA vaccination administered. The above confirm the altered immune cellular response of T1D patients following mRNA vaccination [27]. Thus, the authors conclude that boosting immunization strategies with multiple doses are necessary for patients with T1D [30, 31].

The low rate of response to vaccination against SARS-CoV-2 in some groups of patients may be attributed to abnormalities in their costimulatory mechanisms, such as the PD1-PDL1 pathway, including children and eventually those with diabetes. In fact, Loretelli et al. [32] showed that a significant proportion of patients who recovered from COVID-19 and suffered from post-COVID syndrome presented altered levels of plasma soluble PD1/PD-L1 immune checkpoint axis and impaired cytokine profiles, indicative of a dysfunctional status of T cells with a poor response to SARS-CoV-2 antigens, which may last for months after their hospital discharge. These immune abnormalities may reduce immune response against viral reinfections and the ability to fight pathogens [33].

Moreover, in patients with T2D, a reduced antibody count and lower seroconversion after the use of Sinovac vs Pfizer-BioNTech vaccine was reported [34]. Seroconversion in patients with T2D was lower than that of patients with other high-risk conditions (obesity, chronic lung disease, cardiovascular conditions, cancer) [34] and could be attributed to delayed and/or reduced immune response [35, 36]. The above indicate the need of COVID-19 vaccination of patients, especially those with specific underlying diseases, such as diabetes.

## Safety of different vaccines against SARS-CoV-2

### mRNA vaccine safety in adolescents > 12 years of age

Following comparison of mRNA vaccine safety between adolescents aged 12–16 years and young adults aged 16–25 years, no differences were noted [37].

Usual side-effects were local (86%), or systemic (14%), including fatigue (60%), headache (55%), myalgia (27%), fever (10%, >40 °C: 0.1%), diarrhea (11%), shivering (10%), joint pain (10%), nausea/vomiting (2–3%), lymph node enlargement (0.8%).

Lee SW et al. [38] reported in adolescents >12 years of age the following side-effects after vaccination with an mRNA vaccine: local symptoms such as pain, local tenderness and redness, and systemic such as fatigue, muscle and joint pain, headache and fever. The severity of these symptoms was mostly grade 1 and 2 and less frequently grade 3 and 4 [37, 39].

Regarding COVID-19 vaccination among adolescents >/12 years of age with T1D, Piccini et al. [29] reported that 39 individuals who received two doses of mRNA vaccine did not present any significant effects on glycemic control, in terms of time in range (TIR), mean glucose levels and total insulin daily dose. They presented only mild side-effects and no adverse reactions [29].

### Risk factors for severe side-effects in young adults post mRNA vaccination

In the study by Lee SW et al. [38] including 1403 young adults, aged >21 years from Korea, who were studied for approximately 1 week after the administration of the 1^st^ dose of the mRNA vaccine, less than half (42%) developed mild/severe side-effects grades 3–4. The risk factors associated with mild/severe side-effect development included:

- Young age:
  - 21–30 years of age: OR = 2.49
  - 31–40 years of age: OR = 1.78
  - 41–50 years of age: OR = 1.47
- Female gender had twice the risk for side-effects (OR = 2.16)
- Low Body Weight had 1.6 times higher risk for mild/severe side-effects (OR = 1.61)
- Comorbidities:
  - Diabetes Mellitus had 2.3 times higher risk for side-effects (OR = 2.36).

Following multifactorial logistic regression analysis, young age and female gender were identified as major contributors of the risk for severe side-effects after the 1^st^ dose of the mRNA vaccine [38].

### Rare/Severe side-effects of the COVID-19 vaccines

Among the rare and severe side-effects of COVID-19 vaccines is myocarditis/pericarditis, occurring in 1.24/100.000 doses, predominantly among healthy young people, mostly males, aged <30 years, with increased frequency in those aged 12–24 years (Table 4) [40].

**Table 4:** VAERS reporting rates of verified myocarditis per 1 million mRNA COVID-19 vaccinations (Pfizer-BioNTech and Moderna combined), days 0–7 post-vaccination

	Dose 2 (primary series)		1^st^ booster dose	
Age group	Male	Female	Male	Female
5–11 years	2.5	0.7	0.0	0.0
12–15 years	47.1	4.2	12.9	0.7
16–17 years	78.7	7.4	21.6	0.0
18–24 years	39.3	3.9	13.1	0.6
25–29 years	15.3	3.5	4.4	2.2
30–39 years	7.8	1.0	1.9	0.9
40–49 years	3.3	1.6	0.2	0.6
50–64 years	0.7	0.5	0.4	0.1
>65 years	0.3	0.5	0.7	0.2

*VAERS* Vaccine Adverse Event Reporting System CDC and FDA [49]

### Rare COVID-19 vaccination side-effects among youth

#### Myocarditis & pericarditis

Cases of myocarditis were reported 6–25 days after the 2^nd^ dose of the vaccine. Predominantly healthy youths are affected (70% <30 years of age, mainly males: 65%) with no underlying conditions (Table 4). Symptoms include angina, dyspnea, fever, fatigue, myalgia. Laboratory results show increased troponin, NT-pro-BNT, CRP and ESR levels. Additionally, an abnormal ECG, heart MRI, decreased LVEF (40-50%) and partial LV hypokinesis are noted. These patients are managed with Colchicine, non-steroidal anti-inflammatory drugs (NSAID), IV-IG and Prednisolone [40]. In a report from the CDC, among 192,405,448 persons in the US, receiving mRNA-based COVID vaccines during the period December 2020 to August 2021, there were 1991 cases of myocarditis (0.98 cases/100,000 vaccinated persons), predominantly occurring after the second dose. The median age was 21 years (IQR: 16–31 years), with the highest myocarditis rates in the 12–15 age group and 82% were males. Of them, 96% were hospitalized and in 87% the symptoms subsided before being discharged. They were predominantly managed with NSAIDs [41].

In general myocarditis and pericarditis after COVID-19 vaccination have a mild course, respond to conservative treatment and are less severe than classical or COVID-related myocarditis. The risk of myocarditis following COVID-19 is 1.8–5.6 times higher than that following COVID vaccination and is reduced if the time distance between the two vaccination doses is 8 weeks [42].

#### Possible pathophysiologic mechanisms of myocarditis and pericarditis

Three possible pathophysiologic mechanisms have been implicated in the development of myocarditis or pericarditis after mRNA vaccines [43].

- Molecular mimicry. It is possible that the spike protein of SARS-CoV2 resembles an unidentified protein of the myocardium. Presentation of the spike protein by antigen presenting cells activates autoreactive T cells that bind to both self and non-self antigens and induces myocardium destruction [44].
- Acute toxic effect of spike protein on myocardial cells, which explains the strong association of mRNA vaccines with myocarditis [45].
- Reaction to adjuvant nanoparticles or other components of the vaccine [46].

Young age is associated with the development of myocardial complications as well as increased effectiveness of the vaccine due to a stronger immune system [47]. Male sex has been proven a major risk factor of myocarditis development. It is possible that testosterone increases the viral binding to the myocardial cells and inhibits the anti-inflammatory actions of cytokines (similarly to in vivo experiments in mice with myocarditis, due to the Coxsackie virus) [48].

In conclusion, myocarditis and pericarditis may follow COVID-19 infection, but have been also classified as side-effects of certain COVID-19 vaccines (2.3% for myocarditis) [40–42, 49]. Increased incidence of myocarditis has been observed in male adolescents of 12–17 years of age. However, it must be noted that vaccination in this age group predictably prevents the disease and its complications. Furthermore, the prognosis of vaccine-related myocarditis and pericarditis is highly favorable. Arrhythmias and myocardiopathy after vaccination are extremely rare. Therefore, the advantages of COVID-19 vaccination surpass the disadvantages, and youth should be encouraged to become immunized [40–42, 49].

#### Anaphylaxis

Patients with a history of an immediate allergic reaction to any other vaccines, due to a known polyethelene glycol allergy or injectable therapies should have a precaution to Janssen COVID-19 vaccine and should consult with an Allergist-Immunologist (Table 1) [50].

## Concerns about children and adolescents’ Covid-19 vaccination

### Pros

Immunization of children and adolescents against COVID-19 is a heated topic of discussion in the literature for various reasons. Although children and adolescents suffer from mild clinical presentation of COVID-19 infection, they may present some rare and severe complications, such as MIS-C, myo/pericarditis and Long COVID syndrome [12]. Reportedly, COVID vaccination offers protection against severe infection, as well as MIS-C and Long COVID syndrome [51].

Moreover, as new and highly contagious variants have arisen, a vaccination percentage of >80% is necessary in order to achieve herd immunity. Since the elderly have been broadly vaccinated, the virus circulates in the younger age groups, whose vaccination could contribute to reaching herd immunity [52, 53]. Additionally, the combination of SARS-CoV2 infection and vaccination, termed “hybrid immunity”, seems to confer the greatest protection against COVID-19 infections [54].

Additionally, transmission among children and adolescents has been reported to be increased after school opening [53], increasing with student age. The highest rates were observed among high-school students and the lowest among infants and toddlers.

Finally, lockdown measures against the COVID-19 pandemic caused numerous problems for students and their caregivers [53].

Severe adverse effects of some COVID-19 vaccines for adolescents are very rare (Table 4) and are outweighed by the advantages. Thus, COVID-19 vaccination among children and adolescents is very important for themselves and their community [55].

### Cons

Children and adolescents generally develop milder COVID-19 infection compared to adults, except for those with underlying diseases [15].

Although COVID-19 vaccines reduce disease transmission from vaccinated persons infected by variants, such as delta and omicron, they do not prevent it. Thus, the vaccination of young children 5–11 years old should aim to protect the individual child and not other persons in the family, school, or the community [56].

Another consideration is that, as currently the COVID pandemic seems to subside, the implementation of large-scale immunization programs among children without underlying chronic diseases might have financial implications and could potentially delay the provision of other health care services, such as routine childhood immunization programs [57].

### Parental and health professional concerns about childhood vaccination against COVID-19

In the childhood age group (5–11 years), high levels of hesitancy towards COVID-19 vaccination have been reported among parents and health care professionals. Thus, Miliordos et al. [58] from Greece recorded the attitudes on COVID-19 vaccination of 366 parents. Of them, 48% agreed to vaccinate their child. The predictors of positive vaccination attitudes were pediatrician recommendation and parental vaccination. Of those who did not intend to vaccinate their children, 80% would do so following their pediatrician’s recommendation. Al Rasheedi AT et al. [59] from Saudi Arabia reported that 2/3 of caregivers were not willing to vaccinate their children against COVID-19. Readiness for COVID-19 vaccination was associated with secondary school parental education, previous COVID-19 infection of family members, previous vaccination with 3 doses of the COVID-19 vaccine without side-effects, previous vaccination against influenza and the absence of chronic diseases in their 5–11-year-old children [59].

Regarding health care professionals’ attitudes on COVID-19 vaccination for children <12 years of age, hesitancy among 68.5% of participants has been reported, who suggested to proceed with caution [60].

### COVID-19 vaccination rate of children and adolescents with T1D

A nationwide survey in Italy in 2022 [61] recorded the intention of parents of T1D adolescents (>/12 years) to vaccinate them against COVID-19. The vaccination rate of T1D adolescents was 79.5%, which was significantly higher than that of their healthy peers (62.4%, *p* < 0.01). Specifically, 75% in the 12- 15-year-old group, and 100% in the 16–18- and 19–25-year-old groups were vaccinated. However, 20% of parents were against COVID-19 vaccination and did not allow it. Mild/moderate side-effects (local pain or fever) presented in 70% of vaccinated patients. Only 3 patients had severe adverse events with full recovery; myocarditis (*n* = 1), loss of consciousness requiring ICU admission (*n* = 1), anaphylactic reaction (*n* = 1). Glycemic imbalance was observed in only 2.5% of patients [61]. A US study among parents of children with T1D [62] with a mean age of 7.8 ± 1.7 years and mean HbA1c levels of 8.3 ± 1.5% in the Mid Atlantic and Southwest Regions, reported that higher HbA1c levels were associated with lower intention to vaccinate, with vaccine safety being the main parental concern. Moreover, higher parental vaccination intention was associated with child private insurance (compared to public), and shorter T1D duration [62].

### Is it necessary to vaccinate all children and adolescents against COVID-19?

Regarding the necessity of universal COVID-19 vaccination in childhood and adolescence, Paul and Mishra [63] in a systematic review of 64 articles, found that 43.75% were in favor of the vaccination, 31.25% were against and 25.0% were neutral. In the articles in favor of COVID-19 vaccination, the main reasons cited were: the increasing rate of disease burden, prevention of school closure and its multifaceted consequences, and protection against severe COVID-19 infection. The factors against vaccination included: mild infection in children and adolescents, ethical concerns and legal problems regarding the consent of minors, and finally parental vaccine hesitancy. Finally, vaccine hesitancy was accentuated with reduced transmission of the disease in the community, and perceived protection against MIS-C and long COVID syndrome [63]. In fact, Borchering RK et al. [64] conducted a multi-model aggregation study, analyzing the weekly counts of cases, hospitalizations, and deaths in the United States for a period of 6 months and concluded that expanding vaccination to children 5–11 years old would provide significant direct benefits to this age group and indirect benefits for all age groups, even for more transmissible variants [64].

Moreover, patients with T1D or T2D have been reported to develop more severe clinical outcomes after COVID-19 infection [65] and impaired glycemic control [18, 19]. Besides, no differences were observed between patients with and without T1D regarding local and systemic effects and glycemic abnormalities following COVID-19 vaccination [27]. Therefore, it is concluded that due to the impaired cytotoxic immune response patients with diabetes should undergo COVID-19 vaccination with booster doses [27].

The WHO recommendations in 2022 on COVID-19 vaccination of different population groups were as follows:

- First priority: Vaccination of all individuals aged >/65 years and of all adults belonging to high-risk groups,
- Second priority: Vaccination of children and adolescents aged 5–18 years with serious underlying conditions,
- Third priority: Vaccination of healthy children and adolescents 5–18 years old [66].

Finally, the ADA Advanced Standards of Care 2023 [67] suggest the following regarding vaccination against COVID-19 and other infections in patients with diabetes:

- People with diabetes should be prioritized and offered SARS-CoV2 vaccines.
- Children and adults with diabetes should receive all age-appropriate vaccinations according to recommendations, as preventing infections reduces hospitalizations, but also reduces the risk of acquiring infections, such as COVID-19.
- In patients with diabetes, impaired glycemic control prior to or during COVID-19 admission have been associated with poor outcomes, including mortality [67]

## Conclusions

Adult patients with DM have an increased risk of severe clinical presentation and death due to COVID-19 infection. Although children and adolescents with T1D present with a milder clinical course and disease duration than their adult counterparts, they have an increased risk of developing DKA, especially in the face of pre-existing poor glycemic control. COVID-19 vaccines have proven effectiveness; however, poor immune response to vaccination is observed among patients with T1D, especially those with poor glycemic control and obesity. COVID-19 vaccines are safe, with generally mild adverse effects. Nevertheless, a few rare adverse effects post-COVID-19 vaccination require attention. Myocarditis and pericarditis are the most worrisome, occurring mostly among young males with very good outcomes. Thus, vaccination of all people over 65 years of age is necessary, and of all adults >18 years belonging in the high-risk groups with severe underlying conditions, for the successful management of the pandemic. Lastly, regarding vaccination of children and adolescents, although the course of COVID-19 disease in this age group is mild, they may present with serious complications (myo/pericarditis, MIS-C, long COVID syndrome), at higher rate than rare vaccine adverse effects. For children and adolescents with T1D, maintaining an excellent glycemic control and COVID-19 vaccination of adolescents, particularly those with poor control, are the best measures to prevent DKA as well as rare and serious disease complications during and following COVID-19 infection.
